# Electrochemical Determination of 17-β-Estradiol Using a Glassy Carbon Electrode Modified with α-Fe_2_O_3_ Nanoparticles Supported on Carbon Nanotubes

**DOI:** 10.3390/molecules28176372

**Published:** 2023-08-31

**Authors:** Juliana Costa Rolim Galvão, Mayara da Silva Araujo, Maiyara Carolyne Prete, Vanildo Leão Neto, Luiz Henrique Dall’Antonia, Roberto Matos, Cesar Ricardo Texeira Tarley, Roberta Antigo Medeiros

**Affiliations:** Department of Chemistry, State University of Londrina, Londrina 86057-970, PR, Brazil; juliana.costa.rolim@uel.br (J.C.R.G.); asmayara@uel.br (M.d.S.A.); mayprete@gmail.com (M.C.P.); vanildosln@gmail.com (V.L.N.); luizh@uel.br (L.H.D.); rmatos@uel.br (R.M.); tarley@uel.br (C.R.T.T.)

**Keywords:** iron oxide, estradiol, multiple-walled carbon nanotubes, square wave voltammetry, glassy carbon electrode

## Abstract

In this study, a novel electrochemical assay for determining 17-β-estradiol (E2) was proposed. The approach involves modifying a glassy carbon electrode (GCE) with a nanocomposite consisting of α-Fe_2_O_3_ nanoparticles supported on carbon nanotubes (CNTs)—denoted as α-Fe_2_O_3_-CNT/GCE. The synthesis of the α-Fe_2_O_3_-CNT nanocomposite was achieved through a simple and cost-effective hydrothermal process. Morphological and chemical characterization were conducted using scanning electron microscopy (SEM), Raman spectroscopy, and energy-dispersive X-ray spectroscopy (EDX). The presence of the α-Fe_2_O_3_-CNT film on the GCE surface resulted in an enhanced electrochemical response to E2, preventing electrode surface fouling and mitigating the decrease in peak current intensity during E2 oxidation. These outcomes substantiate the rationale behind the GCE modification. After the optimization of experimental conditions, E2 was determined by the square wave voltammetry technique using 0.1 mol L^−1^ KCl solution (pH = 7.0) with 20% ethanol as a supporting electrolyte. A linear concentration range of 5.0–100.0 nmol L^−1^ and a low limit of detection of 4.4 nmol L^−1^ were obtained. The electroanalytical method using α-Fe_2_O_3_-CNT/GCE was applied for E2 determination in pharmaceutical, lake water, and synthetic urine samples. The obtained results were attested by recovery tests and by high-performance liquid chromatography as a comparative technique at a 95% confidence level. Thus, the developed electrochemical sensor is simple and fast to obtain, presents high accuracy, and is viable for determining E2 in routine analysis.

## 1. Introduction

In the last several years, the effect of endocrine-disrupting compounds in animal and human systems has drawn the attention of scientists, research communities, and the public. They do this by imitating the biological functions of natural hormones, occupying hormonal receptors, and disrupting the transportation and metabolic processes of these natural hormones [1,2]. The 17-β-estradiol hormone (E2) is an estrogen with significant endocrine-disrupting capabilities. It is produced in the ovaries and is responsible for maintaining the menstrual cycle, reproductive system, and lipid metabolism. It also plays a role in the growth and development of the skin, brain, and sinus tissue. Additionally, E2 is present in the testicles and is important for maintaining bone structure and sperm production [3]. Moreover, E2 is also used as a medication for hormone replacement therapy and contraceptives [4].

E2 is excreted daily by humans and released into aquatic environments and sewage effluents along with industrial waste, as water treatment systems do not completely remove this hormone and its derivatives. The bioaccumulation of E2 in fish, birds, and reptiles can lead to immune system complications and reproductive anomalies. When present at abnormal levels in humans, E2 can cause health problems such as ovary cancer, cirrhosis, hyperthyroidism, early puberty, hypertension, and polycystic ovary syndrome [3,5,6,7].

Hence, the detection of E2 using methods with high sensitivity and low detection limits is very important. In the literature, methods for determining E2 mainly employ gas [8] and liquid [9,10,11,12] chromatography. Most of these methods require tedious sample preparation and the analysis is very delayed.

Over the past few decades, electroanalytical methods have arisen as potent substitutes for conventional analysis techniques. The strength of these methods lies in their versatility and advantageous analytical capabilities, offering an array of benefits including cost-effectiveness, heightened sensitivity, portability, and user-friendliness. As a result, many electroanalytical methods stand as viable alternatives for E2 determination, owing to their uncomplicated instrumentation, economical nature, and the absence of intricate sample pre-treatment procedures or the necessity for toxic organic reagents [2].

In these studies, various electrodes are employed, with the majority being chemically modified using a range of nanomaterials. These include materials like wrinkled mesoporous carbon [13], molecularly imprinted polymer [14,15], multi-walled carbon nanotubes and gold nanoparticles [16], gold nanoparticles, graphene, and carbon nanotubes [17], graphene [6,18], graphene quantum dots [19], iron oxide [20,21,22,23], etc. However, some types of modified electrodes have complicated preparation steps and require the usage of expensive solvents or nanomaterials.

Therefore, in the present work, the primary objective is to propose a novel electroanalytical method utilizing a modified electrode with a nanocomposite obtained through a simple, less time-consuming, and cost-effective hydrothermal method.

Electrodes based on carbonaceous materials are widely used because they are inexpensive and readily available, have a broad potential range and low background current, and are chemically inert during electrochemical analysis [24,25]. Additionally, the modification of these electrodes with different materials can improve the selectivity and high detection limits of the electroanalytical methods. As a result, the use of chemically modified electrodes has significantly increased, leading to the need for exploring new materials that provide high surface area, stability, and excellent conductivity [26,27,28,29]. Among the most used materials were metallic oxides [30,31] and carbonaceous nanomaterials such as graphene nanosheets [32,33], and carbon nanotubes (CNTs) [34,35].

CNTs are cylindrical structures produced by the winding of graphene sheets composed of carbon atoms with sp^2^ hybridization in a hexagonal structure. They find wide applications in electrochemistry, primarily due to their conjugated π-bonds, which give them a steric bulkiness like an alkene with electron deficiency. Additionally, this material consists of hexagonal nets with high aromaticity, which enhances conductivity on its surface [36]. Therefore, CNTs are commonly employed in electrode modifications due to their large electrochemical window, excellent conductivity, expansive surface area, and stability, all amplifying electron transfer [17].

α-Fe_2_O_3_, a metal oxide, possesses great utility and finds applications in numerous fields like magnetic storage devices, supercapacitors, photocatalysts, and sensors [37,38]. It possesses advantageous qualities such as non-toxicity, biocompatibility, and affordability. Additionally, the α phase of this semiconductor is formed during the final stage of iron oxide synthesis, contributing to its exceptional chemical stability and resistance to corrosion [39,40]. In the literature, there are examples of the use of the α-Fe_2_O_3_ and CNT composites in the modification of electrodes. They have been primarily used for the electroanalytical determination of drugs [41,42,43].

In this context, the purpose of the present work was a novel assay of electrochemical E2 determination using a glassy carbon electrode (GCE) modified with a nanocomposite of α-Fe_2_O_3_ nanoparticles supported by CNTs, obtained by a simple and inexpensive hydrothermal synthesis.

## 2. Results

### 2.1. Chemical and Morphological Characterization of α-Fe_2_O_3_-CNT/GCE

**Figure 1:**
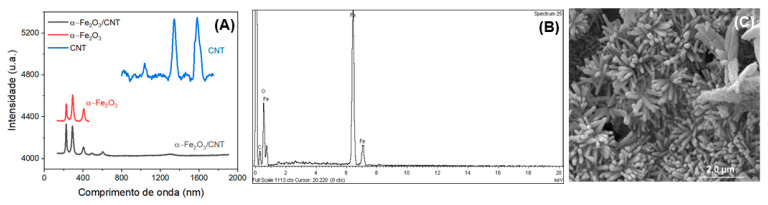
(**A**) Raman spectra of CNT, α-Fe_2_O_3,_ and α-Fe_2_O_3_-CNT; (**B**) EDX spectrum of α-Fe_2_O_3_-CNT; (**C**) SEM image of α-Fe_2_O_3_-CNT.

Raman, SEM, and EDX were used to investigate the successful preparation of α-Fe_2_O_3_/CNT nanocomposite, and the results are shown in Figure 1. As can be seen from Figure 1A, in the Raman spectra of the CNTs the D and G band characteristics of this material for α-Fe_2_O_3_ observed bands located at 227, 294, and 407 cm^−1^ can be well assigned to Eg modes of Fe_2_O_3_ [44]. The Raman spectra obtained for α-Fe_2_O_3_-CNT show only the bands corresponding to Fe-O bonds, characteristic of this iron oxide. It was not possible to observe characteristic bands of carbonaceous materials in the region from 1330 to 1600 cm^−1^. This can be justified by the small region where the laser beam is incident on the materials, performing measurements in areas where no carbonaceous materials were present, and/or by the low concentration of these materials in the hydrothermal synthesis.

In EDX spectra (in Figure 1B), the presence of essentially three elements in the materials: carbon, iron, and oxygen, was observed, as expected [45]. This indicated that the synthesis of this material was satisfactory, and based on the intensities of the bands, a higher percentage of iron and oxygen compared to the carbonaceous material can be noted. This confirmed the initial proportions of the materials used, with a larger amount of the iron precursor when compared with CNTs. The SEM image (Figure 1C) obtained for α-Fe_2_O_3_-CNT showed a material in the form of rods with good homogeneity, and the CNTs were coated with α-Fe_2_O_3_.

### 2.2. Electrochemical Behavior of E2 at α-Fe_2_O_3_-CNT/GCE

**Figure 2:**
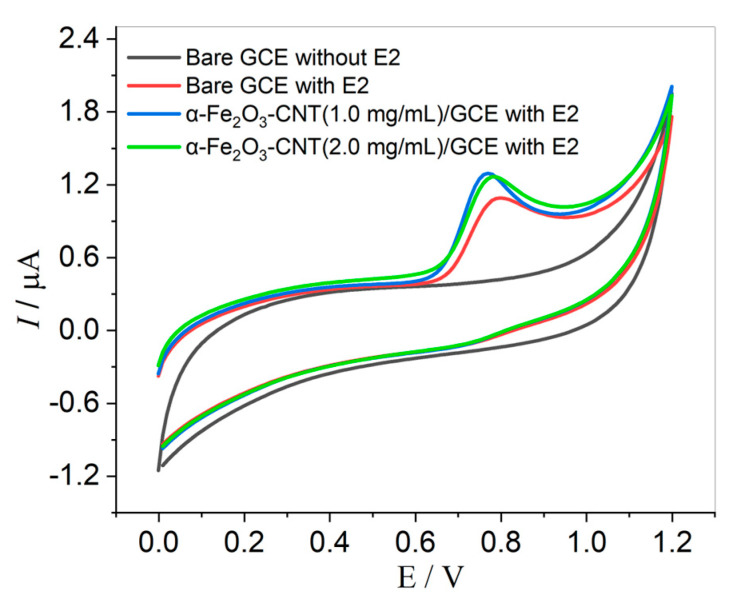
Cyclic voltammograms obtained for 0.1 µmol L^−1^ E2 in KCl solution (10% *v*/*v* ethanol) pH 7.0 using bare GCE, α-Fe_2_O_3_-CNT/GCE (1.0 mg mL^−1^), and α-Fe_2_O_3_-CNT/GCE (2.0 mg mL^−1^). Scan rate: 50.0 mV s^−1^.

Cyclic voltammetry (CV) technique assays were performed in the presence of 0.1 µmol L^−1^ of E2 in a 0.1 mol L^−1^ KCl solution with 10% *v*/*v* ethanol (to prevent the precipitation of E2). Initially, we observed a comparison of the bare GCE and GCE modified with α-Fe_2_O_3_-CNT, and the concentration of α-Fe_2_O_3_-CNT in the suspension also was evaluated, with concentrations of 1.0 and 2.0 mg mL^−1^. As shown in Figure 2, the hormone E2 exhibits a single oxidation peak around 0.8 V, indicating an irreversible electrochemical process. This behavior is consistent with other reports found in the literature regarding E2 determination [6,16,46,47,48].

The voltammograms obtained with the bare GCE for E2 showed a peak with good current intensity. However, after the modification with α-Fe_2_O_3_-CNT, the peak current increased, indicating an improvement in the interaction with the electrode surface and an effective enhancement in the conductivity of the working electrode. This can be attributed to a reduction in the electron transfer resistance and an increase in the electrode surface area. Furthermore, two concentrations were evaluated, and no significant difference in the voltammetric response was observed. Thus, the α-Fe_2_O_3_-CNT suspension at a concentration of 1.0 mg mL^−1^ was adopted for further experiments.

The composition and pH of supporting electrolytes play an important role in the electrochemical response. Therefore, KCl solution (0.1 mol L^−1^), phosphate buffer solution (0.01 mol L^−1^), and Britton–Robinson buffer solution (0.04 mol L^−1^), all at pH 7.0, were evaluated (results not shown). It was observed that the KCl solution provided a peak with higher intensity and a lower oxidation potential when compared to the others. Thus, KCl 0.1 mol L^−1^ was chosen as the supporting electrolyte for the next experiments.

The influence of the pH of the KCl solution was also investigated (see Figure S1 in Supplementary Material). For pH values above 10.0, the E2 oxidation process becomes kinetically unfavorable as it leads to the passivation of the electrode surface. High peak current intensities were obtained at pH 6.0, 7.0, and 8.0. However, the electrochemical response at pH 7.0 exhibited better peak definition and repeatability (RSD of 0.65%; *n* = 3). According to Mustafa et al. (2004) [49], α-Fe_2_O_3_ nanoparticles have a point of zero charge (pH pcz) of 6.50. Conversely, the pK_a_ of the E2 molecule is 10.7. Therefore, at pH 7.0, the E2 molecule is protonated, while the α-Fe_2_O_3_ nanoparticles are negatively charged, resulting in an electrostatic interaction between α-Fe_2_O_3_-CNT/GCE and the E2 molecules.

A linear relationship between peak potential (E_p_) and pH (E_p_(V) = 1.00193–0.05060 pH; R^2^ = 0.9934) was obtained in the pH range of 2.0 to 6.0, where the E_p_ shifts to less positive values as the pH increases. This behavior was described by Vega et al. (2007) [50] as characteristic behavior for the oxidation of phenolic compounds. The slope of 0.0506 V pH^−1^ is close to the theoretical Nernstian slope of 0.0592 V pH^−1^, which indicates that the E2 oxidation process involves the same number of protons and electrons. This result is in accordance with the oxidation mechanism of E2 proposed by Ngundi et al. (2003) [51], in which the oxidation reaction involves transferring two protons and two electrons.

**Figure 3:**
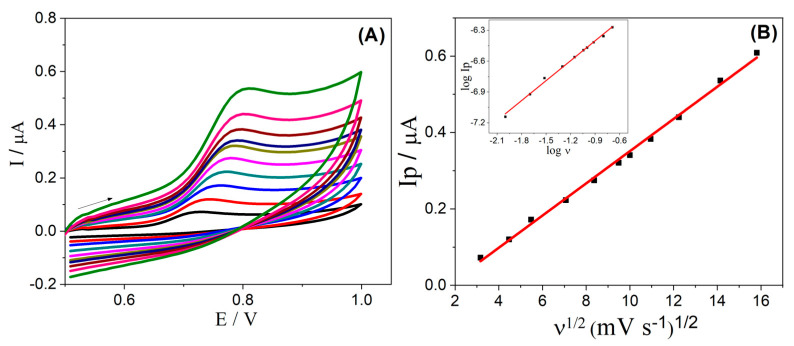
(**A**) Cyclic voltammograms obtained for 0.1 µmol L^−1^ E2 in 0.1 mol L^−1^ KCl solution (10% *v*/*v* ethanol), pH 7.0, using α-Fe_2_O_3_-CNT/GCE at different scan rates (10–250 mV s^−1^). (**B**) Relation of I_p_ versus ν^1/2^; Inset: relation of log I_p_ (μA) versus log (ν^1/2^) (mV s^−1^). The arrow indicates the direction of the potential scan in cyclic voltammetry.

The electrochemical behavior of α-Fe_2_O_3_-CNT/GCE was studied by CV at different scan rates (from 10 to 500 mV s^−1^) in the presence of E2, as can be seen in Figure 3. It was noted that the overpotential was shifted positively (Figure 3A), which is characteristic of an irreversible process. Furthermore, the relation of I_p_ versus ν^1/2^ shown in Figure 3B reveals a linear behavior, and the slope of 0.6 obtained in the relation of log I_p_ versus log ν (inset Figure 3B) is close to the theoretical value of 0.5 for a diffusion-controlled process; these results confirm a diffusion-controlled process of species from solution to the electrode surface [52].

The results obtained in the scan rate study were also used to determine the number of electrons involved in the oxidation process of the E2 molecule through Equation (1):

|E_p_ − E_p/2_| = 47.7 mV/αn (1)

where E_p_ is the anodic peak potential, E_p/2_ is the potential associated with the peak current half-height, α is the charge-transfer coefficient (0.50), which is pre-determined for organic molecules, and *n* is the number of electrons consumed in the reaction [52,53].

**Figure 4:**
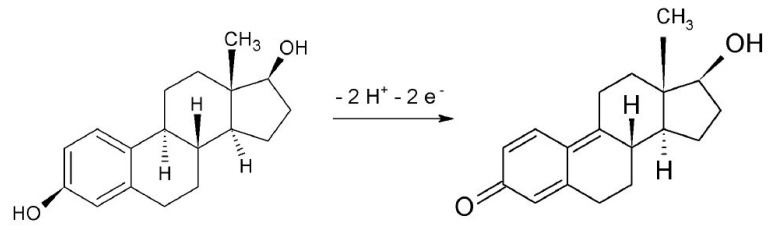
Reaction mechanism proposed for E2 oxidation on the α-Fe_2_O_3_-CNT/GCE surface.

Considering the mean value of E_p_ = 0.769 V and E_p/2_ = 0.709 V obtained with CV assays in different scan rates, the value of *n* calculated was 1.6. This result is in accordance with the oxidation mechanism reported by Ngundi et al. (2003) [51], where the oxidation reaction involves the transfer of two electrons and two protons, as shown in Figure 4.

### 2.3. Determination of E2 at α-Fe_2_O_3_-CNT/GCE

**Figure 5:**
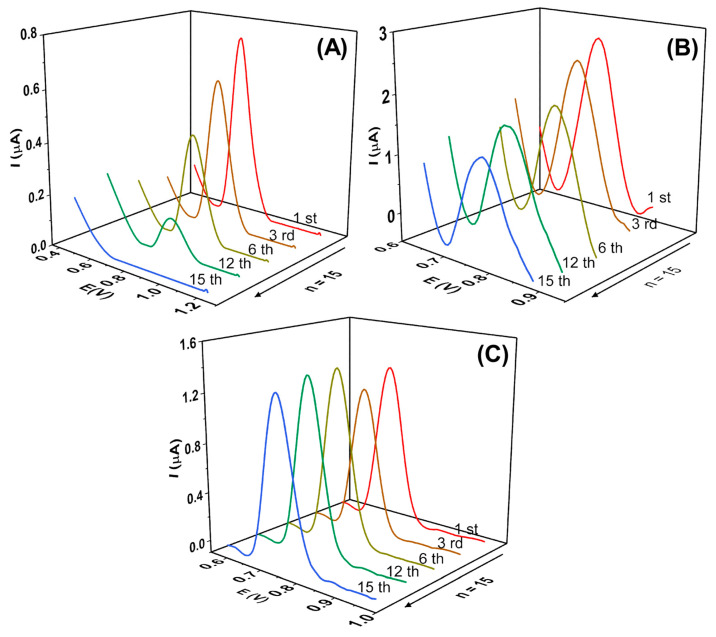
Square wave voltammograms obtained for 0.1 µmol L^−1^ E2 using (**A**) bare GCE, (**B**) CNT/GCE (1.0 mg mL^−1^), (**C**) α-Fe_2_O_3_-CNT/GCE. Supporting electrolyte: 0.1 mol L^−1^ KCl solution (10% *v*/*v* ethanol) pH 7.0, SWV parameters: (ꬵ) = 60 s^−1^; (a) = 40 mV, and (ΔEs) = 6 mV.

As reported in the literature, it is common for the E2 electrochemical oxidation process to involve the adsorption of the E2 molecule or its oxidation products on the electrode surface. This can result in a decrease in I_pa_ (peak current) and reduced precision of the analytical method [6]. Therefore, this phenomenon was evaluated using the square wave voltammetry (SWV) technique with the bare GCE, CNT/GCE, and α-Fe_2_O_3_-CNT/GCE. A total of 15 consecutive measurements were conducted in the presence of 0.1 µmol L^−1^ E2 in KCl solution (10% *v*/*v* ethanol) at pH 7.0, as shown in Figure 5.

As can be seen for the bare GCE, a significant decrease in peak current intensity was observed, with a relative standard deviation (RSD) of 71%. Furthermore, the peak potential shifted toward more positive values as the measurements were taken, indicating passivation of the GCE surface and a slower interaction between the electrode surface and the E2 molecule. When the GCE electrode was modified with CNT, it was observed that the variation was lower compared to the bare GCE, with an RSD of 26%. On the other hand, when the α-Fe_2_O_3_-CNT/GCE was used, the RSD was 4%, indicating that the modification of the GCE surface significantly improved the electrode performance by preventing fouling and enhancing the analytical signal.

The SWV technique parameters were optimized as follows: frequency (*ꬵ*) (10–100 s^−1^), pulse amplitude (*a*) (10–150 mV), and scan increment (ΔE_s_) (1–12 mV). The responses were evaluated in terms of peak definition, repeatability, and peak current intensity. The selected values were *ꬵ* = 60 s^−1^, *a* = 40 mV, and ΔE_s_ = 6 mV.

**Figure 6:**
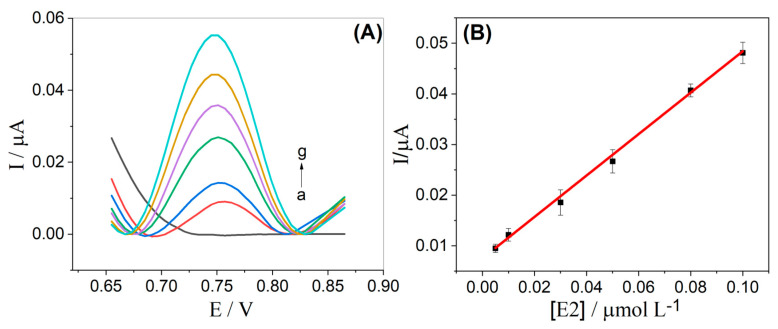
(**A**) SW voltammograms obtained for E2 in different concentrations: (a) blank; (b) 5.00; (c) 10.00; (d) 30.00; (e) 50.00; (f) 80.00; and (g) 100.00 nmol L^−1^; (**B**) Analytical curve obtained for E2, using an α-Fe_2_O_3_-CNT/GCE. Supporting electrolyte: 0.1 mol L^−1^ KCl solution (10% *v*/*v* ethanol) pH 7.0. SWV parameters: (*ꬵ*) = 60 s^−1^; (*a*) = 40 mV and (ΔE_s_) = 6 mV.

After this previous study, the analytical curve was obtained, and SWV voltammograms were carried out with successive additions at concentrations of E2 ranging from 5.0 to 100.0 nmol L^−1^, as shown in Figure 6A. The peak current obtained for E2 exhibits a linear dependence on the respective concentration (see Figure 6B) and is described by the equation: I_p_ (μA) = 0.0072 μA + 0.4083 μA μmol^−1^ L [E2] (μmol L^−1^) (R^2^ = 0.998).

The limit of detection (LOD) was calculated as 3 std/m, where std represents the standard deviation of 10 determinations of the blank and m is the slope of the analytical curve. The obtained LOD value was found to be 4.4 nmol L^−1^. Next, the values of intra- and inter-day repeatability were obtained for two concentrations of E2 (10.0 and 50.0 nmol L^−1^). For the intra-day repeatability experiments, ten successive measurements (n = 10) were obtained on the same day, while for the inter-day repeatability measurements, they were obtained over five successive days (n = 3). The obtained RSD values ranged from 3.8% to 8.6%, indicating good precision of the proposed method.

The analytical parameters obtained in this proposed method were compared with other electroanalytical methods for the determination of E2 using different electrochemical sensors reported in the literature. As shown in Table 1, the method proposed here presents similar or smaller LOD values compared to most works found in the literature. The significant advantage of the proposed method lies in its relatively low cost and simple synthesis of the material.

**Figure 7:**
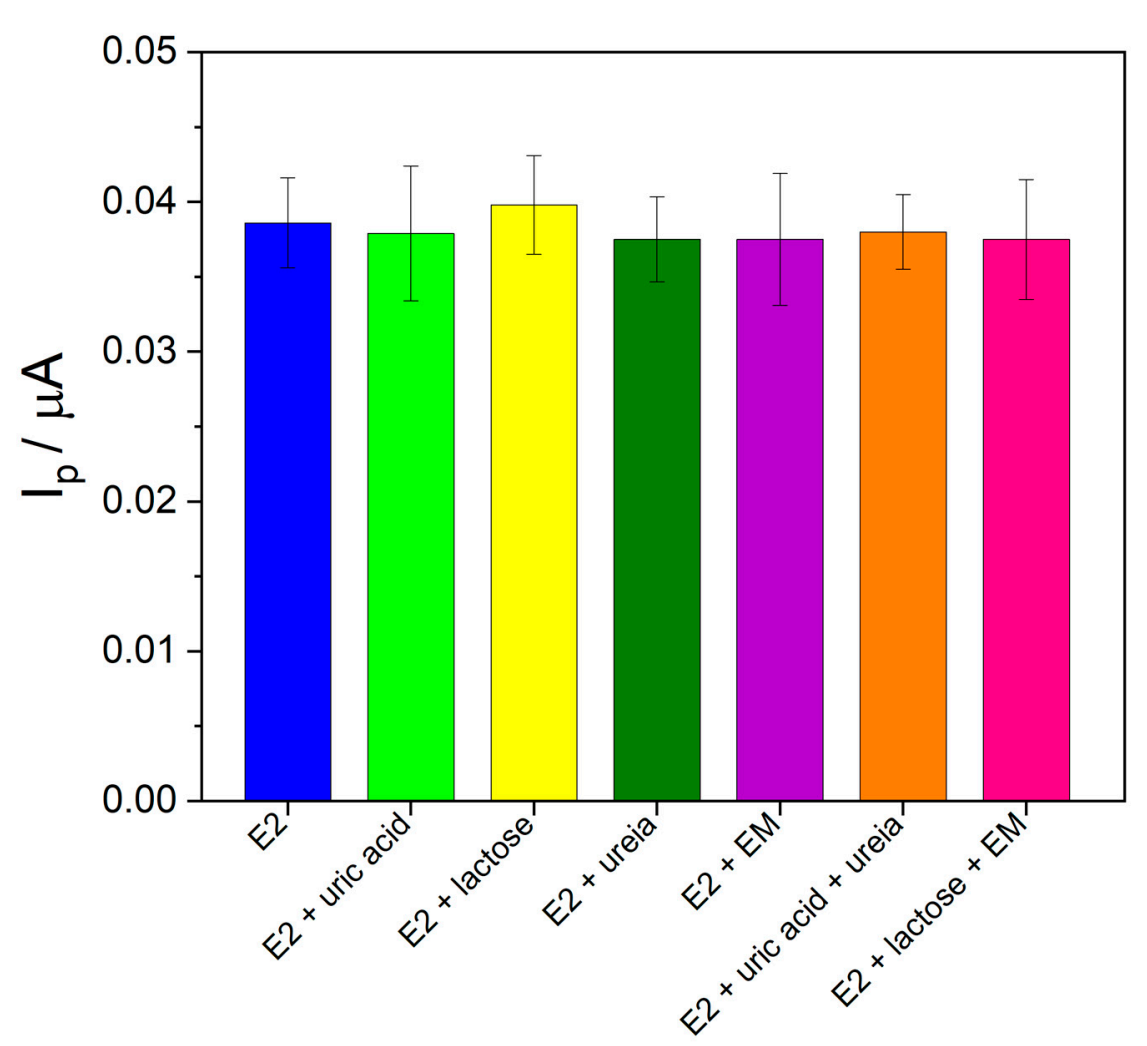
Bar diagram of the interference effect study for E2 determination.

To evaluate the effect of possible interfering molecules found in the pharmaceutical and urine samples, binary solutions were prepared containing E2 with uric acid, lactose, urea, or magnesium stearate (at a ratio of 1:10 (*m*/*m*) E2:interfering molecule). Additionally, solutions containing E2 with a mixture of interfering molecules (uric acid + urea or lactose + magnesium stearate) were studied at the same ratio of 1:10 (*m*/*m*) E2:interfering molecule mixture. As can be seen in Figure 7, the current intensity obtained for E2 in the presence of the interfering molecules was compared to the current obtained for E2 alone under the same experimental conditions. The relative errors ranged from 1.50% to 3.22%, indicating that the evaluated molecules did not interfere with the determination of E2 under the studied conditions.

It is important to emphasize that other estrogens such as estradiol, estrone, and estriol may also be present in biological and environmental samples [57]. These hormones oxidize at the same potential as E2, making them potential interference species. Therefore, in the presence of other estrogens, it is only possible to determine the total estrogen concentration in the samples.

To evaluate the matrix effect of lake water and synthetic urine samples on E2 determination using α-Fe_2_O_3_-CNT/GCE, addition–recovery studies were conducted. For this purpose, the samples were spiked with 50 nmol L^−1^ of E2. The recovery values are presented in Table 2, and they were found to be 105% for the lake water sample and 100% for the synthetic urine sample. These results suggest that the proposed method is applicable for determining E2 in real urine samples. Moreover, it can also be employed for water analysis using samples containing a higher E2 concentration, or for water samples subjected to E2 preconcentration treatment, as no matrix effects were observed.

Finally, the proposed electroanalytical method was used for the determination of E2 in commercial pharmaceutical samples. Two samples were evaluated, and the results obtained by the proposed method were statistically compared with the results obtained by the high-performance liquid chromatography (HPLC) method. The E2 concentrations found in the pharmaceutical samples for both methods are presented in Table 3. A low relative error (%) was observed for both samples, demonstrating the accuracy of the method. Additionally, the Student’s *t* test was used to compare the results, yielding experimental t values of 1.2 for sample 1 and 1.6 for sample 2, both of which were lower than the critical t value (t_critical_ = 2.8). This indicates that there is no significant difference between the proposed method and the HPLC comparative method, at a 95% confidence level.

## 3. Materials and Methods

### 3.1. Chemical Reagents and Solutions

All the chemical reagents used in this work were of analytical grade. Boric acid (H_3_BO_3_), acetic acid (CH_3_COOH), phosphoric acid (H_3_PO_4_), uric acid (C_5_H_4_N_4_O_3_), 2-propanol (CH_3_)_2_CHOH), sodium sulfate (Na_2_SO_4_), potassium dihydrogen phosphate (KH_2_PO_4_), sodium phosphate (Na_3_PO_4_), disodium hydrogen phosphate (Na_2_HPO_4_), multiwalled carbon nanotubes (CNTs), Fe_2_O_3_, polyvinylpyrrolidone (PVP) and sodium nitrite (NANO_3_) were obtained from Sigma-Aldrich; potassium chloride (KCl), sodium chloride (NaCl), hydrochloric acid (HCl), calcium chloride (CaCl_2_·2H_2_O), and sodium hydroxide (NaOH) were obtained from Sinth.

Solutions were prepared using ultrapure water (18.2 MΩ cm) from an ELGA^®^ PURELAB Maxima (Woodridge, IL, USA) purification system. Pharmaceutical tablet samples were purchased from a local drugstore. E2 stock solution was prepared in ethanol at 0.1 mol L^−1^ concentration, then, proper dilutions in ultrapure water were performed. For the preparation of the BR buffer solution (0.01 mol L^−1^), appropriate amounts of boric acid, phosphoric acid, and acetic acid were mixed and dissolved in ultrapure water. The phosphate buffer solution was prepared by weighing adequate amounts of KH_2_PO_4_ and Na_2_HPO_4_ and dissolving them in ultrapure water. The 0.1 mol L^−1^ KCl solution was prepared by dissolving its salts in ultrapure water. The pH of phosphate and BR buffer and KCl solution was adjusted using 2.0 mol L^−1^ NaOH solution and 1.0 mol L^−1^ HCl solution. All the solutions were stored in a refrigerator until use.

### 3.2. Apparatus

Voltammetric measurements were carried out using a potentiostat/galvanostat PalmSens 2.0 (PalmSens BV, Houten, Netherlands) driven by PStrace 5.3 software using a conventional three-electrode electrochemical cell containing the bare GCE or the α-Fe_2_O_3_-CNT/GCE as working electrodes, an Ag/AgCl (3.0 mol L^−1^ KCl) as reference electrode, and a platinum wire as auxiliary electrode. For the morphological and chemical characterization a scanning electronic microscope (model FEI Quanta 200) and a DeltaNu spectrometer were used. The determination of E2 by a comparative HPLC method was carried out using a high-performance liquid chromatograph (model Shimadzu LC-20AD) equipped with a UV-Vis detection system using a detector of diode array (purchased in Tokyo, Japan), a C18 column (model Phenomenex) at 250 mm × 4.5 mm in dimension and particle size of 5 μm.

The quantification of E2 was performed according to the procedure described by Yilmaz and Kadioglu (2013) [58] with some modifications: a mobile phase in isocratic mode consisting of methanol and water (70:30 *v*/*v*), a flow rate of 1.0 mL min^−1^, injection volume of 20.0 μL, UV-Vis detection at 220 nm. Before injection in the chromatographic system, the samples and standards were filtered through a 0.25 µm PTFE membrane filter. The pH control in the preparation of solutions was performed using a Bench Top Water pH meter (model AZ86505).

### 3.3. Synthesis of α-Fe_2_O_3_-CNT and Preparation of Electrochemical Sensors

The synthesis of α-Fe_2_O_3_ nanoparticles supported by carbon nanotubes was achieved by mixing 3.0 mg mL^−1^ of CNT, 3.0 g of Fe_2_O_3_, and subsequently adding 6.0 g of NaNO_3_, 0.45 g of PVP, and 80.0 mL of ultrapure water. The mixture was then stirred for 30 min. Subsequently, it was transferred to a Teflon autoclave, and the hydrothermal process was carried out at 160 °C for 12 h. After the reaction, the solid product was filtered, washed with ethanol and ultrapure water, dried in an oven at 80 °C for 12 h, and finally calcined using a muffle furnace at 600 °C for 3 h, resulting in the formation of the metallic oxide [59].

The α-Fe_2_O_3_-CNT suspensions were prepared at concentrations of 1.0 mg mL^−1^ and 2.0 mg mL^−1^. In both suspensions, the polyelectrolyte dihexadecyl hydrogen phosphate (DHP) was added at a concentration of 1.0 mg mL^−1^, and the mixture was dispersed in ultrapure water. The CNT suspension was prepared using 1.0 mg mL^−1^ of CNT and 1.0 mg mL^−1^ of DHP, also in ultrapure water. To achieve homogeneity, the suspensions were subjected to 2 h of ultrasonic treatment and 1 h of magnetic stirring. The polyelectrolyte DHP acts as a surfactant and forms a stable film on the electrode surface when dispersed in water under ultrasonic stirring [60].

Before the modification procedure, the surface of the GCE was polished using 3.0 μm alumina suspension. After washing, a second polishing was performed using fine-grit sandpaper and water. Subsequently, the electrode was subjected to a 3-min ultrasonic bath in 2-propanol, followed by another 3-min ultrasonic bath in ultrapure water. The cleaning and conditioning process was completed by carrying out an electrochemical treatment in a sulfuric acid solution with a concentration of 0.01 mol L^−1^ using the CV technique. The treatment involved 50 cycles in a potential range of −0.5 to 1.2 V and a scan rate of 100 mV s^−1^. The GCE was modified by drop-casting 6 μL of the aqueous suspensions of CNT or α-Fe_2_O_3_-CNT (at concentrations of 1.0 or 2.0 mg mL^−1^) onto its surface. Subsequently, the modified electrode was left to dry at room temperature for a minimum of 2 h.

### 3.4. Preparation of Lake Water, Synthetic Urine, and Pharmaceutical Samples

Natural water samples were collected at Igapó Lake in Londrina, Paraná, Brazil, using a clean amber glass bottle. Then, the samples were filtered through qualitative filter paper and used in the preparation of a 0.1 mol L^−1^ KCl solution (pH = 7.0), which was used as a supporting electrolyte. Aliquots of E2 were added to the water sample and quantified by the addition and recovery test.

The synthetic urine sample was prepared according to the procedure described by Laube, Mohr and Hesse [61] as follows: 0.110 g of CaCl_2_·2H_2_O, 0.296 g of NaCl, 0.225 g of Na_2_SO_4_, 0.140 g of KH_2_PO_4_, 0.160 g of NH_4_Cl, and 2.5 g of urea were dissolved in 100 mL of ultrapure water. For analysis, 1.0 mL of synthetic urine sample was diluted in 19 mL of 0.1 mol L^−1^ KCl solution (pH = 7.0) with 10% ethanol. Aliquots of E2 were added to the urine sample and quantified by the addition and recovery test.

For the pharmaceutical sample preparation, ten tablets were weighed and ground in a mortar with a pestle; then, the amount of one tablet was weighed and diluted in 5.0 mL of ethanol, then for E2 determination was used an external calibration method.

### 3.5. Measurement Procedure

CV and SWV were employed to investigate the electrochemical behavior and the quantification of E2. The instrumental parameters for SWV were optimized, and the respective analytical curve was obtained by adding small volumes of concentrated standard solutions of the E2 to the supporting electrolyte solution. The limit of detection (LOD) value was calculated as three times the standard deviation for 10 measurements of the blank solution (s) divided by the slope of the respective analytical curve (b) (LOD = *3* s/b) [62]. The repeatability of the electroanalytical methods was checked with intra-day (n = 10) and inter-day (n = 5) determinations for two different concentrations of E2, for which the respective relative standard deviations (RSDs) were calculated.

The selectivity of the proposed methods was evaluated by the addition of possible interferents present in pharmaceutical formulations and urine samples (uric acid, urea, lactose, starch, povidone) to a standard solution containing E2, in the concentration ratios (standard solution to interferent) of 10, 1, and 0.1.

## 4. Conclusions

This work describes the successful development of an electrochemical method based on a glassy carbon electrode modified with α-Fe_2_O_3_ nanoparticles supported on CNTs. We explored the high electroactive surface area of CNTs and the high conductivity of α-Fe_2_O_3_ nanoparticles. The synthesis of the α-Fe_2_O_3_-CNT nanocomposite was achieved through a simple and cost-effective hydrothermal process. The α-Fe_2_O_3_-CNT/GCE provided an increase in current intensity obtained for E2 electrochemical oxidation and prevented fouling on the electrode surface when compared with a bare GCE. After optimizing the experimental conditions and SWV technique parameters, E2 determination was performed within a linear concentration range, and a LOD in the nanomolar order was achieved.

The proposed α-Fe_2_O_3_-CNT/GCE proved to be a simple and fast method for E2 determination in lake water, synthetic urine, and pharmaceutical samples, with good precision, accuracy, sensitivity, and no matrix effect or interference from other molecules. It can be a less expensive alternative for routine determinations of these drugs.

## Figures and Tables

**Table 1:** Comparison of results obtained for determination of E2 by the here-proposed method and by other electrochemical methods reported in the literature.

Sensor	Technique	Linear Range (mol L^−1^)	LOD (mol L^−1^)	Reference
RGO-CuTthP/GCE ^a^	DPV	1.0 × 10^−7^–1.0 × 10^−6^	5.3 × 10^−9^	[6]
wMC/GCE ^b^	DPV	5.0 × 10^−8^–1.0 × 10^−5^	8.3 × 10^−9^	[13]
MMIP/MCPEc ^c^	DPAdSV	6.0 × 10^−8^–1.8 × 10^−4^	2.0 × 10^−8^	[14]
mag-MIP/GEC ^d^	SWV	5.0 × 10^−8^–7.5 × 10^−4^	1.0 × 10^−8^	[15]
AuNP-MWCNT/GCE ^e^	LSV	1.0 × 10^−6^–2.0 × 10^−5^	7.0 × 10^−9^	[16]
rGO-AuNPs/CNT/SPE ^f^	DPV	5.0 × 10^−8^–1.0 × 10^−6^	3.0 × 10^−9^	[17]
Ab-Au-RG-SPCE ^g^	EIS ^j^	0.0–1.2 × 10^−7^	1.5 × 10^−9^	[18]
LAC-CP/Pt ^h^	CV	1.0 × 10^−7^–1.2 × 10^−4^	9.9 × 10^−7^	[19]
LAC-GQDs/Au ^i^	CV	5.0 × 10^−6^–5.0 × 10^−5^	1.5 × 10^−6^	
CuO/CPE ^j^	SWV	6.0 × 10^−8^–8.0 × 10^−7^	2.1 × 10^−8^	[47]
AuNP-Thi-CNTs/GCE ^k^	DPV	1.2 × 10^−11^–6.0 × 10^−8^	1.5 × 10^−12^	[48]
Fe_3_O_4_-NC/GCE ^l^	DPV	1.0 × 10^−8^–2.0 × 10^−5^	4.9 × 10^−9^	[20]
Fe_3_O_4_ NPs-BMI.PF6/CPE ^m^	SWV	1.0 × 10^−7^–1.0 × 10^−6^	5.0 × 10^−8^	[21]
HRP-Pol/Pt ^n^	DPV	1.0 × 10^−7^–2.0 × 10^−4^	1.0 × 10^−7^	[54]
Fe_3_O_4_-MIP/SPCE ^o^	SWV	5.0 × 10^−8^–1.0 × 10^−5^	2.0 × 10^−8^	[22]
Fe_3_O_4_-MIP@RGO/GCE ^p^	DPV	5.0 × 10^−8^–1.0 × 10^−5^	8.2 × 10^−10^	[23]
MWCNT/GCE ^q^	SWV	2.5 × 10^−7^–1.0 × 10^−5^	1.0 × 10^−8^	[55]
CuPc-P6LC-Nafion/SPEF ^r^	DPV	8.0 × 10^−8^–7.3 × 10^−6^	5.0 × 10^−9^	[56]
Fe_2_O_3_-CNT/GCE	SWV	5.0 × 10^−9^–1.0 × 10^−7^	4.4 × 10^−9^	This work

^a^ RGO-CuTthP/GCE: Glassy carbon modified with Cu(II)-meso-tetra(thien-2-yl) porphyrin supported over reduced graphene oxide; ^b^ wMC/GCE: Glassy carbon electrode modified with wrinkled mesoporous carbon (wMC) nanomaterial; ^c^ MMIP/MCPEc: Magneto carbon paste electrode based on magnetic molecularly imprinted polymer; ^d^ mag-MIP/GEC: Graphite–epoxy composite electrode modified with magnetic nanoparticles (mag; Fe_3_O_4_) coated with molecularly imprinted polymers (MIPs); ^e^ AuNP-MWCNT/GCE: Glassy carbon electrode modified with multi-walled carbon nanotube and gold nanoparticle; ^f^ rGO-AuNPs/CNT/SPE: Screen-printed electrode modified with gold-nanoparticle-decorated reduced graphene oxide–carbon nanotubes; ^g^ Ab-Au-RG-SPCE: Screen-printed carbon electrode modified with electro-reduced graphene and porous gold structure; ^h^ LAC-CP/Pt: Platinum electrode modified with poly [4-(5-hexylthiophen-2-yl)-2,6-bis(5-(selenophen-2-yl)thiophen-2-yl)pyridine] (conducting polymer) and laccase; ^i^ LAC-GQDs/Au: Gold electrode modified with graphene quantum dots and laccase; ^j^ CuO/CPE: Carbon paste electrode modified with Copper (II) oxide; ^k^ AuNP-Thi-CNTs/GCE: Glassy carbon electrode with carbon nanotubes, thinine and gold nanoparticles; ^l^ Fe_3_O_4_-NC/GCE: Glassy carbon electrode modified with Fe_3_O_4_-doped nanoporous carbon; ^m^ Fe_3_O_4_ NPs-BMI.PF6/CPE: Carbon paste electrode modified with Fe_3_O_4_ magnetite nanoparticles and 1-butyl-3-methylimidazolium hexafluorophosphate ionic liquid; ^n^ HRP-Pol/Pt: Platinum electrode modified with polymer-poly(4,7-bis(5-(3,4-ethylenedioxythiophene)thiophen-2-yl)benzothiadiazole) and horseradish peroxidase; ^o^ Fe_3_O_4_-MIP/SPCE: Screen-printed carbon electrode modified with magnetic molecularly imprinted polymer; ^p^ Fe_3_O_4_-MIP@RGO/GCE: Glassy carbon electrode modified with Fe_3_O_4_ nanobeads immobilized on graphene; ^q^ MWCNT/GCE: Glassy carbon electrode modified with a multi-walled carbon nanotube; ^r^ CuPc-P6LC-Nafion/SPEF: Screen-printed electrode made of carbon ink modified with copper phthalocyanine (CuPc), Printex 6L carbon P6LC and nafion film.

**Table 2:** Addition and recovery study for E2 determination in lake water and synthetic urine samples using α-Fe_2_O_3_-CNT/GCE.

Sample	Added (µmol L^−1^)	Found (µmol L^−1^)	Recovery (%) ^a^
Lake water	0.05	0.052 ± 0.005	105
Synthetic urine	0.05	0.050 ± 0.007	100

^a^ Recovery (%) = ([Found]/[Added]) × 100%; n = 3.

**Table 3:** Results obtained for E2 determination in commercial pharmaceutical samples by the proposed SW voltammetric method and the high-performance liquid chromatography (HPLC) comparative method.

	E2 Concentration (mg/Tablet)			
Sample	Labeled	HPLC Method	Proposed Method	Relative Error (%) ^a^
1	1.0	0.96 ± 0.02	0.95 ± 0.05	1.0
2	2.0	1.83 ± 0.03	1.81 ± 0.02	1.1

^a^ RE (%) = [(Proposed method content) − (HPLC method content)/(HPLC method content)] × 100%.

## Data Availability

The datasets used and/or examined during the present study can be obtained by contacting the corresponding author through a reasonable request.

## Supplementary Materials

The following supporting information can be downloaded at: https://www.mdpi.com/article/10.3390/molecules28176372/s1, Figure S1: Cyclic voltammograms obtained for 0.1 mmol L^−1^ of E2 using α-Fe_2_O_3_-CNT/GCE at different pHs (A) Ep versus pH plot (B). Supporting electrolyte: 0.1 mol L^−1^ KCl solution (10% *v*/*v* ethanol) in the presence of 0.1 µmol L^−1^ E2. Scan rate: 50 mV s^−1^.
