# Supplementary material for: Electrochemical Determination of 17-β-Estradiol Using a Glassy Carbon Electrode Modified with α-Fe2O3 Nanoparticles Supported on Carbon Nanotubes
Source: Molecules. 2023 Aug 31;28(17):6372. doi: 10.3390/molecules28176372 (PMC10489867; doi:10.3390/molecules28176372)
Supplement: Supplementary file 1 [file molecules-28-06372-s001.zip › molecules-2556540-supplementary.pdf]

# Supplementary Material

## Electrochemical Determination of 17-β-Estradiol Using a Glassy Carbon Electrode Modified with α-$Fe_2O_3$ Nanoparticles Supported on Carbon Nanotubes

**Juliana Costa Rolim Galvão, Mayara da Silva Araujo, Maiyara Carolyne Prete, Vanildo Leão Neto, Luiz Henrique Dall'Antonia, Roberto Matos, Cesar Ricardo Texeira Tarley and Roberta Antigo Medeiros ***

Department of Chemistry, State University of Londrina, Londrina 86057-970, PR, Brazil; juliana.costa.rolim@uel.br (J.C.R.G.); asmayara@uel.br (M.d.S.A.); mayprete@gmail.com (M.C.P.); vanildosln@gmail.com (V.L.N.); luizh@uel.br (L.H.D.); rmatos@uel.br (R.M.); tarley@uel.br (C.R.T.T.)

* Correspondence: ramedeiros@uel.br; Tel.: +55-43-33714811

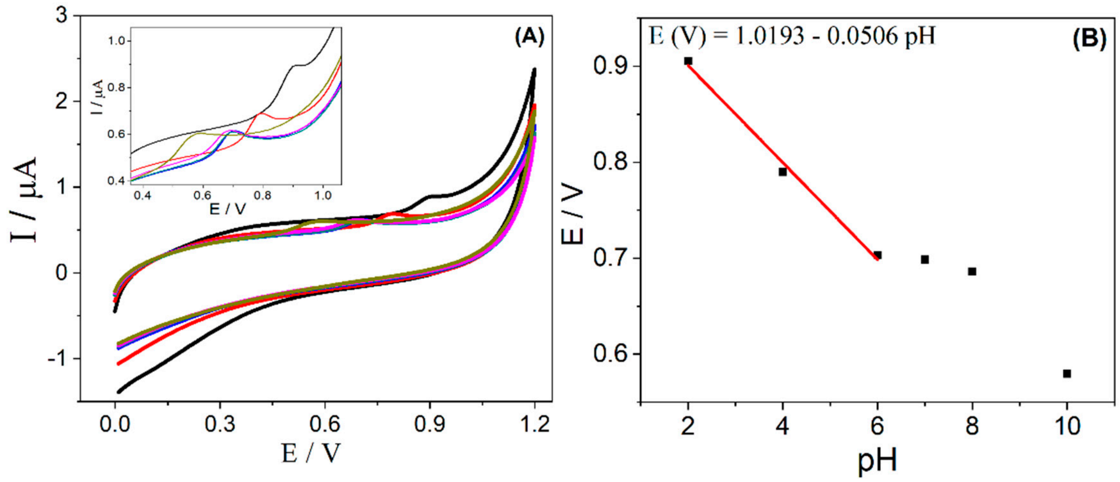


**Figure S1:** Cyclic voltammograms obtained for 0.1 mmol L$^{-1}$ of E2 using $\alpha$-$Fe_2O_3$-CNT/GCE at different pHs (A) $E_P$ versus pH plot (B). Supporting electrolyte: 0.1 mol L$^{-1}$ KCl solution (10% v/v ethanol) in the presence of 0.1 µmol L$^{-1}$ E2. Scan rate: 50 mV s$^{-1}$.
